# Nurse-delivered sleep restriction therapy in primary care for adults with insomnia disorder: a mixed-methods process evaluation

**DOI:** 10.3399/BJGP.2023.0162

**Published:** 2023-12-29

**Authors:** Stephanie Armstrong, Julie Pattinson, Aloysius Niroshan Siriwardena, Simon D Kyle, Peter Bower, Ly-Mee Yu, Yaling Yang, Emma Ogburn, Nargis Begum, Leonie Maurer, Barbara Robinson, Caroline Gardner, Victoria Lee, Dimitri Gavriloff, Colin A Espie, Paul Aveyard

**Affiliations:** Community and Health Research Unit, School of Health and Social Care, University of Lincoln, Lincoln, UK.; Community and Health Research Unit, School of Health and Social Care, University of Lincoln, Lincoln, UK.; Community and Health Research Unit, School of Health and Social Care, University of Lincoln, Lincoln, UK.; Sleep and Circadian Neuroscience Institute, Nuffield Department of Clinical Neurosciences, University of Oxford, Oxford, UK.; National Institute for Health and Care Research School for Primary Care Research, Centre for Primary Care and Health Services Research, University of Manchester, Manchester, UK.; Nuffield Department of Primary Care Health Sciences, University of Oxford, Oxford, UK.; Nuffield Department of Primary Care Health Sciences, University of Oxford, Oxford, UK.; Nuffield Department of Primary Care Health Sciences, University of Oxford, Oxford, UK.; Nuffield Department of Primary Care Health Sciences, University of Oxford, Oxford, UK.; Mementor DE GmbH, Leipzig, Germany.; Nuffield Department of Primary Care Health Sciences, University of Oxford, Oxford, UK.; Centre for Primary Care and Health Services Research, University of Manchester, Manchester, UK.; Centre for Primary Care and Health Services Research, University of Manchester, Manchester, UK.; Sleep and Circadian Neuroscience Institute, Nuffield Department of Clinical Neurosciences, University of Oxford, Oxford, UK.; Sleep and Circadian Neuroscience Institute, Nuffield Department of Clinical Neurosciences, University of Oxford, Oxford, UK.; Nuffield Department of Primary Care Health Sciences, University of Oxford, Oxford, UK.

**Keywords:** cognitive behavior therapy, general practice, insomnia disorder, nursing primary care, programme evaluation

## Abstract

**Background:**

Sleep restriction therapy (SRT) is a behavioural therapy for insomnia.

**Aim:**

To conduct a process evaluation of a randomised controlled trial comparing SRT delivered by primary care nurses plus a sleep hygiene booklet with the sleep hygiene booklet only for adults with insomnia disorder.

**Design and setting:**

A mixed-methods process evaluation in a general practice setting.

**Method:**

Semi-structured interviews were conducted in a purposive sample of patients receiving SRT, the practice nurses who delivered the therapy, and also GPs or practice managers at the participating practices. Qualitative data were explored using framework analysis, and integrated with nurse comments and quantitative data, including baseline Insomnia Severity Index score and serial sleep efficiency outcomes to investigate the relationships between these.

**Results:**

In total, 16 patients, 13 nurses, six practice managers, and one GP were interviewed. Patients had no previous experience of behavioural therapy, needed flexible appointment times, and preferred face-to-face consultations; nurses felt prepared to deliver SRT, accommodating patient concerns, tailoring therapy, and negotiating sleep timings despite treatment complexity and delays between training and intervention delivery. How the intervention produced change was explored, including patient and nurse interactions and patient responses to SRT. Difficulties maintaining SRT, negative attitudes towards treatment, and low self-efficacy were highlighted. Contextual factors, including freeing GP time, time constraints, and conflicting priorities for nurses, with suggestions for alternative delivery options, were raised. Participants who found SRT a positive process showed improvements in sleep efficiency, whereas those who struggled did not.

**Conclusion:**

SRT was successfully delivered by practice nurses and was generally well received by patients, despite some difficulties delivering and applying the intervention in practice.

## Introduction

Insomnia disorder, characterised by persistent problems with sleep initiation and/or maintenance and daytime consequences, is associated with mental and physical conditions and significantly impaired quality of life.^1^ Affecting 10%–12% of adults, it is the commonest sleep disorder and the second most prevalent mental health complaint in Europe.^2^ The economic impact of insomnia is high because of the direct and indirect costs associated with increased healthcare usage, absenteeism, reduced productivity (‘presenteeism’), and accidents.^3^

Current guidelines recommend cognitive behavioural therapy for insomnia (CBT-I) as the first-line treatment,^4^^,^^5^ but lack of access to CBT-I in primary care^6^ means GPs provide sleep hygiene advice, hypnotics, or sedative antidepressants in preference.^7^ Sleep restriction therapy (SRT), a key ingredient of CBT-I, involves restricting and standardising a patient’s time in bed to address key perpetuating factors, including excessive or irregular time in bed, and daytime napping.^8^

Meta-analysis suggests that SRT can reduce insomnia symptoms,^9^ but most trials have been performed in specialist research settings. In one small randomised controlled trial (RCT), a brief version of SRT significantly reduced insomnia severity compared with sleep hygiene alone at 6 months (Cohen’s *d* = 0.54) when delivered in two sessions by a GP to a highly selected group of patients with insomnia, free from comorbidities or medication use.^10^ The need for a pragmatic trial testing a scalable model of treatment delivery in primary care, with a representative sample of people with insomnia, led to the design of the Health-professional Administered Brief Insomnia Therapy (HABIT) trial.

HABIT was a parallel, open-label RCT to determine the effectiveness of nurse-delivered SRT compared with sleep hygiene alone in adults (aged ≥18 years) meeting criteria for insomnia disorder recruited within primary care.^11^ Participants randomised to SRT received instructions from the nurse to keep a sleep diary, implement an agreed bed and rise time, and calculate their sleep efficiency before each consultation. Patients received one consultation per week for 4 weeks, adjusting bed and rise times (sleep window) depending on reported improvements in sleep efficiency.^11^ Nurse-delivered SRT was found to be clinically effective in reducing insomnia symptoms and improving sleep-related quality of life, depressive symptoms, mental health-related quality of life, and work productivity at 3-, 6-, and 12-months follow up.^12^ SRT was also highly likely to be cost-effective at a cost-effectiveness threshold of £20 000 per quality-adjusted life-year gained.^12^

**Table:** How this fits in

Insomnia is the commonest sleep disorder and the second most prevalent mental health complaint in Europe with high economic costs because of health use, absenteeism, and reduced productivity. Lack of access to cognitive behavioural therapy for insomnia (CBT-I), which is the recommended first-line treatment for insomnia, led to the development and evaluation, through a randomised controlled trial, of sleep restriction therapy (SRT), a component of CBT-I, delivered by primary care nurses. This process evaluation of the Health-professional Administered Brief Insomnia Therapy (HABIT) trial of SRT with sleep hygiene compared with sleep hygiene alone, showed that SRT was successfully implemented with high fidelity by nurses and positively received by patients despite some initial difficulty adapting to changes in sleep schedules.

The current study is a process evaluation of the HABIT intervention, in line with UK Medical Research Council (MRC) guidelines, to understand intervention delivery, fidelity, and acceptability from the perspective of patients, practice nurses, and other stakeholders.^13^ The aim was to explore how nurse-administered SRT in primary care worked, by examining implementation, mechanisms of impact, and contextual factors. ‘Implementation’ explores how the intervention is delivered and what is delivered, including the training and resources available as well as fidelity of delivery and any adaptations made. ‘Mechanisms of impact’ explores the impact on participants, including perceived benefits and unintended or adverse effects. Exploring ‘contextual factors’ affecting implementation can help to understand the potential for sustaining and scaling the intervention more widely.

## Method

### Design

A mixed-methods design was used, integrating qualitative interview data and quantitative data collected from intervention participants. Data collection and analysis were conducted before the effectiveness and cost-effectiveness outcomes of the trial were revealed.

### Qualitative interviews

Semi-structured interviews were undertaken with patients who had received SRT, nurses delivering the intervention, and the practice managers or GPs at participating practices, using separate interview schedules (see Supplementary Boxes S1–S3).

The aim was to interview 15 patients, five in each of the three trial areas: Oxford, Manchester, and Lincolnshire. Patients were asked during their baseline assessment appointment for consent to be interviewed. Interviewees were selected from those consenting who had completed SRT within 6 months of interview, allowing exploration of how participants felt at various stages of the intervention. All nurses who delivered the intervention were contacted by the research team following consent and interviewed to explore their perceptions of providing SRT. Finally, practice managers or GPs from each participating practice were invited for interview, and those who consented were asked about their perceptions of the impact on the practice, and the sustainability and scalability of the intervention. Interviews were conducted by telephone and were digitally recorded and transcribed.

### Quantitative data

Patient interviewees’ perceptions of the intervention were compared with two quantitative measures, baseline Insomnia Severity Index (ISI) and sleep efficiency recorded at each intervention session. ISI is a seven-item self-reported questionnaire, scoring between 0 and 28, which assesses the severity, nature, and impact of insomnia,^14^ whereas sleep efficiency is the percentage of time in bed spent asleep (0%–100%), which typically increases in participants for whom SRT is successful. Self-reported sleep efficiency was measured with a sleep diary over 7 days at baseline, and during the nurse intervention (recorded in nurse notes for treatment sessions 2–4). Sleep efficiency was also measured at baseline using actigraphy in all participants and in a proportion of participants at 3- and 6-month follow up. Fidelity assessments of audiotaped consultations were conducted by a clinical psychologist (one of the authors independent of the initial analysis) using a bespoke rating system and expressed as a percentage score.^11^

### Data analysis and integration

Qualitative data were examined using framework analysis supported by NVivo (version 12). Two researchers conducted interviews and checked transcripts. Through familiarisation with the transcripts, examination of the interview schedules, and the three MRC framework domains, a set of a priori categories were developed to form an initial framework (Box 1).

**Box 1.** Framework of categories and codes

**Process evaluation, key theme**	**Category**	**Nurse codes**	**Patient codes**	**Practice manager /GP codes**
**Implementation of SRT**	Delivery of intervention	Consultations Modification to delivery Planned delivery Scaling of intervention Worksheet paperwork	Delivery as expected Well explained What could be improved? Positive effects Post-consultation Understanding	Logistics Staff attitudes Wider implementation
	HABIT trial training	Improvement		GP experience of treating insomnia
		Positives Quality Refresher training		GP understanding of intervention Overall experience of the trial
	Patient expectations		Concerns Expectations of SRT Previous experiences	
**Mechanisms of impact of SRT**	Response of patient	Barriers End of therapy Initial response Logistics Patient attitude Withdrawal	Comparison to other treatments Effects on patients Feelings of patients Improvements in insomnia Maintain SRT after trial	
**Contextual factors in providing SRT**	Contextual factors	Previous experience Other	Challenges to SRT Face-to-face appointments Interactions with nurse Telephone appointments Other	Other

*HABIT = Health-professional Administered Brief Insomnia Therapy. SRT = sleep restriction therapy.*

Transcripts were coded independently (by the same two authors). An ‘other’ category was included in the framework to include relevant data that did not readily fit into the pre-existing categories. Although categories applied to each of the groups interviewed (nurse, patient, and practice manager/GP), the codes were specific to each group as outlined in Box 1. Three researchers, one of whom was independent of the initial analysis, discussed and agreed the final themes presented in the results.

Quantitative measures were presented at baseline (ISI and sleep efficiency) and at the nurse follow-up appointments (sleep efficiency) to show changes during treatment. Relationships between qualitative findings, nurse records, and quantitative measures were explored and presented using a joint display (see Supplementary Table S1) allowing for direct comparison of the patient perceptions of SRT with changes to their measured sleep efficiency.

## Results

In total 16 patients, 13 nurses, six practice managers, and one GP were interviewed. Patients were aged from 19 to 74 years (mean 56 years, standard deviation 15 years), seven were male and nine were female, and all identified as White British. Patients are designated by region (A, B, and C), sex (M or F) and age in years, for example, Patient AF57.

Nurses are designated by number and whether they were a clinical research network nurse (CRN nurse) or practice nurse (Nurse). As a result of a lack of availability of nurses at specific practices, two regions utilised research nurses (employed by their local CRN rather than practice nurses). Local CRN research nurses covered >1 practice and therefore 13 nurse participants were interviewed.

In two regions, practices formed consortia under one management group, so seven interviews were undertaken in the practice manager (six interviews) or GP (one interview) category.

Themes are listed under implementation, mechanisms of impact, and contextual factors.

### Implementation of SRT

The implementation category captured themes related to how the intervention was delivered, what was delivered, and what the patients expected.^13^

#### Patients’ expectations due to lack of experience of behavioural therapy

Patients did not know what to expect from SRT as most had no previous experience of behavioural therapy:

*‘No, it was the only sort of formal treatment I’ve had. I’ve tried things like relaxation, and things like that, but this was the only sort of scientific treatment I’ve had.’*

(Patient AM57)

Patients hoped for improvements in their sleep pattern and daytime symptoms. They expressed how uncomfortable they felt if they had not slept well:

*‘I had the hope, rather than the expectation, that it would make me feel better in the morning; I would feel fresh, less tired.’*

(Patient CF19)

#### Feeling prepared, flexible appointment times, and preferring face-to-face

Nurses felt prepared and were supported with adequate training and tools enabling them to deliver SRT effectively:

*‘It was quite straightforward, and obviously we were provided with a PowerPoint presentation to go through; so that was really helpful.’*

(Nurse 13)

Nurses and patients highlighted appointment flexibility as important. Face-to-face appointments were important for the initial appointment and maintaining motivation:

*‘So, I had a one-to-one meeting with a nurse, and I felt that those are really beneficial for me in terms of maintaining that treatment. For me personally, I don’t think I would have done it without the one to one.’*

(Patient AM57)

*‘You absolutely can’t do the first one on the phone.* [Although] *From a patient perspective, it’s very convenient I guess, because they don’t have to come back to the practice.’*

(CRN nurse 1)

#### Accommodating concerns and tailoring therapy

When patients had difficulty implementing SRT, particularly where their routines had an impact on intervention delivery, nurses were able to modify SRT to the patient:

*‘There’s only one really out of the three* [participants]*, where I think there was a bit more tweaking of the times, if you like, and changing; purely because their routine was different.’*

(CRN nurse 1)

*‘We tried to come up with a bit of a solution to it because not everybody is the same, so I felt it would be easier for me if I could knock it off in the morning. So, I didn’t mind getting up at 5.30 am rather than staying up* [later the night before]*.’*

(Patient AF63)

SRT required individuals to calculate their sleep efficiency, which was challenging for some patients. Nurses tailored sessions according to the patient’s ability to comprehend the process:

*‘It varied definitely. Some patients were able to engage very quickly, and the sessions could be done within 20–25 minutes because the patients were well engaged, able to understand the maths, able to understand what we wanted from them, how it was going to influence their sleep. Other patients, however, were very surprised about what they were expected to do, finding the concept very difficult.’*

(Nurse 5)

#### Negotiating sleep timings

Nurses negotiated bed and rise times with patients, as the protocol allowed for minor amendments to SRT to support a patient-centred approach:

*‘And I said I was really struggling to get up at 5 am in the morning at the moment. So, we moved that to 5:15 am last week.’*

(Patient LNF51)

*‘I have actually played around with it* [flexibility in sleep times] *if they had been over 85%* [for sleep efficiency]*; particularly as I have got more used to it. I think initially when you start something; you get worried about how strict you have to be.’*

(Nurse 9)

#### Learning to deliver despite complexity of SRT

Initially, delivery and understanding of SRT involved a learning curve for both patients and nurses, who often adopted a collaborative approach to learning:

*‘It was a learning curve for both the nurse and myself; between us we worked out what was needed.’*

(Patient CF65)

Nurses felt that the intervention was easy to deliver with practice:

*‘I think I was probably quite nervous to start with; but I think that is probably like most things, something new, and you do have teething problems when you start anything new.’*

(Nurse 2*)*

Patients felt that they were able to calculate sleep efficiency but suggested that simplifying this might help retain people on the intervention:

*‘I expect other people would drop out because it took them a lot of time doing the calculations.’*

(Patient AF55)

*‘It is very difficult to explain maths over the phone to a patient if they really struggle to understand it.’*

(Nurse 5)

#### Challenge of delays

For some nurses there were delays between training and seeing their first SRT patient, which increased the challenge of delivery:

*‘I think that was difficult because to do training and then wait, like quite a long time, till you are actually, physically seeing patients.’*

(Nurse 4)

In one case nurses paired up to deliver SRT for their first patient to boost their confidence. This minor divergence from the protocol was agreed in advance and might have been problematic if subsequent sessions were delivered by different nurses:

*‘I think myself and* [another nurse] *doing it together, we seem to work quite well at this point, but as we get more patients, I think both of us will feel confident enough to do it on our own.’*

(Nurse 10)

### Mechanisms of impact of SRT

The study also explored causal mechanisms, specifically how the delivered intervention produced change. The authors were interested in how participants interacted with nurses and responded to SRT and its effects.^13^ This was crucial to understanding how the intervention worked.

#### Self-motivation and effort

Nurses observed that self-motivated patients were more likely to continue with SRT during and after the intervention period and those that put in the effort were more likely to succeed:

*‘The patients that have made it to the end of the study* [end of intervention delivery]*, have taken it upon themselves to continue that process at home. I think it is because the patients that have made it through are self-motivated patients.’*

(CRN nurse 1)

#### Difficulty changing sleep habits

Some patients tried hard to adhere with their SRT but changing their existing sleep habit was challenging:

*‘I had to stay up till midnight, and I thought – I’m never going to be able to do this. And I tried my hardest, but that was very difficult for me actually.’*

(Patient AF63)

#### Experiencing anticipated benefits

Most patients reported that the initial week could be hard but, after that, they started to feel the benefits. They felt more refreshed, their sleep efficiency increased, and they were able to fall asleep more quickly and stay asleep:

*‘I mean after you get over that initial first week, you start to feel the benefits of it. I mean physically it hasn’t helped, because my condition, there’s not a cure for, but mentally I’m so much better for it, and it’s worth sticking with and seeing it through.’*

(Patient CF64)

Patients noted they fell asleep more quickly than before SRT and spent less time in bed awake. Nurses observed that patients receiving SRT perceived bedtime as a more positive experience and there were changes in the perception of sleep:

*‘Frequently could be anything up to an hour or an hour and a half previously, but now down to 15, 20, 25 minutes maximum, most nights before I drop off.’*

(Patient CM65)

*‘She wasn’t having a nap, it was becoming a positive thing because she was looking forward to going to bed; and knowing that when she went to bed, she’d sleep. And even if she woke up, she said, she might wake up once or twice in the night, but she was able to get straight back off to sleep again. So that was good.’*

(CRN nurse 1)

#### Continuing support for adverse effects

Patients did report some adverse effects during the initial phases of SRT:

*‘But the second week and third week, I felt exhausted. Really exhausted, so. Then felt alright again this week.’*

(Patient CF51)

*‘I feel I could do with going to bed a bit earlier. I know in the booklet it suggests that you do things, but when you are so tired, you just can’t function.’*

(Patient CF73)

Several patients and nurses highlighted the need for continued support following the end of the 4-week therapy:

*‘Well for me anyway, 4 weeks wasn’t long enough for me at all … What’s the point? So, I have reverted back to having naps now in the day. So, my insomnia at night has got worse.’*

(Patient BF60)

*‘They were a bit like — “Where do we go from now?” … The chap I think was a little bit — “Oh!” ; a little bit lost, if anything — “What do I do now?”; because he has not got anyone to report to at the end of the week. So just reassuring that he would get follow up at 3 months, 6 months.’*

(Nurse 3)

#### Difficulties maintaining SRT

Some patients expressed difficulties with very early rise times and the ability to maintain SRT every day:

*‘My hardest bit was the getting up at the time she wanted me to get up; and I couldn’t do that. I was getting up way too early, way too early.* [that is, too early as far as this patient was concerned or comfortable with].*’*

(Patient BF60)

#### Reasons for withdrawal

Nurses shared opinions about why patients were likely to withdraw from the intervention, which was related to conflicting commitments, tiredness, negative attitudes (in particular, where there was perceived to be an impact on other commitments), and lack of self-efficacy:

*‘*[Patients might say:] *Well, I can’t do it on a Saturday because of this, and I can’t do this or that.’*

(CRN nurse 1)

*‘That was the biggest complaint that he just felt far too tired and didn’t feel he could go about his daily routines and things because of the tiredness.’*

(Nurse 2)

### Contextual factors in providing SRT in primary care

Practice managers, GPs, and nurses all commented on contextual factors, relating to the practicalities of delivery within GP practices and the facilitators and challenges of sustaining and scaling up the intervention more widely.^13^

#### Time constraints and conflicting priorities for nurses

Practice managers were aware that nurses had concerns about time constraints. These included the difficulties of fitting in extended SRT appointments into existing consultation times that were generally shorter. There were also concerns about prebooking the appointments in advance, again because of lack of time:

*‘The nurse practitioners who are doing the study, they are enjoying doing it, but they are worried about time constraints; and in particular trying to get those four appointments booked in on a weekly basis. And in general practice, that’s very difficult for us.’*

(Practice manager 2)

#### Freeing up GP time

SRT could free up GP time, because it was an intervention that might stop patients calling into the surgery for sleep medication or to discuss their sleep problems:

*‘I think this is something that GPs would take on board quite readily because actually it’s taking work away from GPs and it’s giving an intervention that will actually free up time, I think, actually free up GP time. So if we can avoid patients phoning in for sleeping tablets, or coming to discuss sleep problems, and sort of following up these patients that goes on and on.’*

(GP)

#### Alternative delivery options

Practice staff felt it would be helpful to designate specific times and days for the SRT clinic to be held. This would help staff organise clinics, book patients for appointments, and free up time for nurses to complete additional administrative tasks associated with SRT delivery. One suggestion was to consider treating SRT like other behaviour change clinics, including using set weekly times:

*‘The way I see it running is, if we treat it like a behaviour change intervention, just like our weight management courses.’*

(Practice manager 5)

Several practice managers wondered about using other staff members such as healthcare assistants:

*‘We have a very capable HCA* [healthcare assistant]*, who would be more than capable of actually sitting and going through this with someone; and obviously that would be a lot more cost effective.’*

(Practice manager 2)

Small group therapy sessions were also suggested as a means of delivery and a way of optimising nurse time:

*‘I think it probably is good for the patients as well because as a group meeting for that education and going through it, there’s like a bit of a support group there for them as well.’*

(CRN nurse 1)

*‘… for me I think a group environment with a nurse would have been just as effective as the one to one.’*

(Patient AF57)

Practice staff, including GPs, were supportive but had reservations about time constraints, availability, and set days for clinics. To ensure the intervention could be delivered in routine general practice, suggestions were made that SRT should be delivered in the format of other behavioural interventions (for example, smoking cessation and weight management).

### Quantitative results and joint display

Most patients interviewed either had an improvement or at least no deterioration in sleep efficiency. Supplementary Table S1 provides a joint display of baseline ISI and baseline and subsequent sleep efficiency data together with summary extracts from any notes made by the nurses during the SRT sessions and a ‘representative’ quote from each patient regarding the SRT process. This indicated, not unexpectedly, that participants who found SRT a positive process were more likely to show improvements in sleep efficiency, whereas those that struggled with SRT did not. Fidelity of nurse delivery was found to be high throughout the trial by the independent reviewer (audiorecordings for session 1, median percentage fidelity score 100% [IQR 96–100], and for session 3, median percentage fidelity score 87.5%, [IQR 75–100]). For full details see Kyle *et al*.^12^

## Discussion

### Summary

In this process evaluation of a trial of SRT with sleep hygiene compared with sleep hygiene alone it was found that SRT, despite its complexity, was successfully implemented with high fidelity by nurses and positively received by patients despite some initial difficulty adapting to changes in sleep schedules. It was found that bed and rise times had to be negotiated and agreed with patients to enable these to be accepted and applied.

### Strengths and limitations

This was a process evaluation of, to the authors’ knowledge, the largest pragmatic clinical trial of psychological therapy for insomnia to date delivered in primary care. A limitation of this study is that the participants interviewed had mostly completed all four SRT sessions. The authors did, however, speak to one participant who left the intervention midway because of an underlying health condition. A better understanding of why people withdrew from the intervention might inform changes to the intervention and ongoing support, leading to better retention.

### Comparison with existing literature

Patients and nurses reported that they were able to quickly grasp the purpose of SRT and related processes. Patients preferred face-to-face consultations and felt that these helped maintain motivation. Although face-to-face interactions have been found to be preferred in some studies, overall the evidence is lacking that therapeutic alliance, disclosure, empathy, attentiveness, or participation differs in face-to-face compared with telephone delivery of psychological interventions.^15^ Some patients found calculating sleep efficiency difficult and felt that they needed help from the nurse, and nurses pointed out that helping someone with calculations over the telephone was harder than in person.

All patients interviewed found the first week of therapy difficult because of reduced time in bed and strict bed and rising times. This is consistent with previous evaluations of SRT, where participants reported worsening of daytime functioning in the first week with improvements felt after a period of adjustment.^16^^,^^17^ Previous research has shown that restriction of time in bed out-performs fixed bed and rise times without restriction.^18^^,^^19^ This suggests that although the initial increase in side effects is challenging, they may also be a necessary part of the therapy linked to the need to harness homeostatic sleep pressure.^20^^,^^21^

In this study there was negotiation between the nurses and the patients regarding sleep times and the need for flexibility, which was supported to some extent by the protocol.^11^ Changing ingrained behaviours, in this case fixed night-time (or daytime nap) routines, was challenging, and the flexibility on the part of the nurses allowed patients to feel some level of control. The flexibility was built into the protocol, and so did not affect the fidelity of delivery. One nurse interviewed did mention sharing delivery of the intervention with a colleague for one patient, which would only have been problematic if the patient was given inconsistent advice.

Participants reported adverse effects such as increased tiredness, ‘exhaustion’, and worries about driving, which have been found in other studies.^17^^,^^21^ Others reported sleep disturbance because of menopause and use of sleep aids (for example, sedatives) that affected allocated sleep time, which should be considered in future rollouts of SRT.

### Implications for research and practice

Patients that did have improved sleep efficiency also reported concerns, most commonly that 4 weeks of SRT was not long enough. All participants found the first week of the intervention very difficult as their body adjusted to limited time in bed. By the third week some were seeing significant benefits. For example, one participant who had improvements in sleep efficiency spoke of being woken by their alarm for the first time in years. Others only started to see benefits by the final week and as such felt the loss of support at the end of the intervention had a direct impact on their motivation to continue. Those that saw improvements earlier expressed being more likely to continue after treatment, whereas those that felt benefits later were more likely to revert to previous habits. One patient reported taking a nap in the afternoon the day after the final session and that they quickly reverted to their previous habits as there was no-one ‘watching over them’. This is a significant finding that indicated the importance of individual, personalised delivery with regular checks continuing for some until new habits and sleep patterns were reinforced. It may be relevant to think about cost-effective refinements to SRT based on these findings, such as extending SRT weekly sessions beyond 4 weeks.

Previous research has shown that it was possible for a GP to deliver an adapted version of SRT in general practice,^10^ and this current study confirmed that it was possible for practice nurses to consistently deliver the intervention. Practice managers and GPs also agreed that the intervention could be successfully delivered by nurses in this setting, which they considered may free up time for GPs. Suggestions to facilitate wider roll out included setting up specific clinics at set times that could be run by healthcare assistants, and running small group sessions like other behaviour change clinics (for example, smoking cessation or weight loss), but this would require further evaluation.

In conclusion, SRT can be successfully delivered by nurses in general practice and was generally well received by patients. Ongoing support after the initial intervention period should be assessed to determine whether this leads to improved adherence and outcome.

## Data

The data are not publicly available due to restrictions arising from ethical permissions for the study.
